# Cultured Meat: A Multidimensional Review of Technological, Nutritional, Ethical, and Regulatory Advances (2020–2025)

**DOI:** 10.1111/1750-3841.70915

**Published:** 2026-02-13

**Authors:** Ana Carolina Agne Ferreira Zão, Wesclen Vilar Nogueira, Filipe Soares Rondan, Priscila Tessmer Scaglioni

**Affiliations:** ^1^ Programa de Pós‐Graduação em Ciência e Tecnologia de Alimentos Universidade Federal do Rio Grande do Sul Porto Alegre Brazil; ^2^ Programa de Pós‐Graduação em Engenharia e Ciência de Alimentos Universidade Federal do Rio Grande Rio Grande Brazil; ^3^ Escola de Química e Alimentos Universidade Federal do Rio Grande Rio Grande Brazil

**Keywords:** alternative proteins, cell‐based food, food safety

## Abstract

Global food systems face increasing environmental, ethical, and health‐related challenges, prompting the search for sustainable protein alternatives. Cultured meat has emerged as a promising option, offering potential benefits such as reduced environmental impact, improved animal welfare, and nutritional customization. However, large‐scale implementation remains limited by technological, economic, ethical, and social constraints. This scoping review synthesizes recent scientific literature (2020–2025) addressing the technological, nutritional, regulatory, ethical, and consumer acceptance dimensions of cultured meat. The analysis integrates multidisciplinary findings to identify major trends, research gaps, and challenges to the transition from laboratory development to market readiness. Persistent technological hurdles include optimizing cell culture conditions, scaffold design, and bioreactor scalability. The nutritional composition, particularly protein and lipid content, often lags behind conventional meat and requires improvement to meet dietary and sensory expectations. Regulatory frameworks remain inconsistent worldwide, with few markets approving commercial sales. Ethical debates continue over animal‐derived inputs and product “naturalness.” Consumer acceptance is influenced by psychological, cultural, and demographic factors, with greater acceptance among younger, educated, and health‐conscious individuals. Ensuring long‐term viability will require standardized safety regulations, cost‐effective production systems, and transparent consumer engagement. Cultured meat represents a transformative innovation with the potential to reshape global protein production, but its success depends on interdisciplinary strategies that balance sustainability, safety, ethics, and public trust.

## 1 Introduction

Global population growth has driven an increase in meat production. According to data from the Food and Agriculture Organization of the United Nations (FAO), per capita meat consumption in 2022 (considering the sum of beef, pork, and poultry) varied significantly across continents in 2022. The Americas had the highest per capita consumption, exceeding 91 kg per person per year, followed by Oceania and Europe, both with an average consumption of approximately 74 kg. In contrast, Asia and Africa had lower average consumption, at around 34 and 14 kg, respectively (FAO 2023b).

Conversely, livestock production, particularly extensive livestock farming, is responsible for the emission of gases with a global warming potential greater than that of carbon dioxide, which contradicts the goals established by the Intergovernmental Panel on Climate Change (IPCC), a scientific‐political organization established in 1988 by the United Nations Environment Programme and the World Meteorological Organization; for example, in 2023, global methane (CH_4_) emissions from livestock, specifically from non‐dairy cattle, were approximately 58.5 kt, with Brazil leading these emissions at about 12.7 kt (FAO 2023c). The IPCC warns that, to avoid potentially catastrophic events, global temperature rise must be limited to 1.5°C (IPCC (Painel Intragovernamental de Mudanças Climáticas) 2018). Given this scenario, it is necessary to devise strategies that meet the growing global demand for more sustainable food models. In this context, cultured meat emerges as a promising alternative to minimize the negative impacts associated with conventional animal farming. Cultured meat is obtained from stem cells or other cell types extracted from live animals and grown in a controlled environment using nutrient‐rich medium to promote growth (Galland and Pacheco 2022).

The main advantages of cultured meat are related to environmental sustainability, animal welfare, and human health. From a public health perspective, intensive livestock systems are major reservoirs for zoonotic pathogens and antimicrobial‐resistant (AMR) bacteria, driven by high stocking densities, routine antimicrobial use, and complex animal–human–environment interfaces, which can transmit pathogens through food chains, direct contact, and environmental exposure; such risks vary by species and production system, with poultry and swine often exhibiting higher prevalence of resistant *Escherichia coli* and *Salmonella* compared to cattle, reflecting divergent husbandry practices and antimicrobial use patterns across sectors. AMR not only threatens human therapeutics but also undermines animal health and welfare by reducing treatment efficacy and increasing disease burdens in herds and flocks (Olaru et al. 2023; Mediouni et al. 2025).

Environmentally, livestock production significantly contributes to greenhouse gas (GHG) emissions, land degradation, water pollution, and biodiversity loss, with beef systems particularly associated with high methane emissions and extensive land use for grazing and feed crops, and pig and poultry operations linked to waste‐related air and water quality issues that affect nearby communities and ecosystems (Parlasca and Qaim 2022; Lianou et al. 2017). Moreover, poor welfare conditions—including confinement, stress, and limited natural behaviors—are associated with compromised immune function and heightened disease susceptibility, which, in turn, can increase antimicrobial demand and further exacerbate both AMR and environmental contamination through waste outputs (Kozajda et al. 2024; Olaru et al. 2023; Trinchera et al. 2025).

By decoupling meat production from live animal rearing, cultured meat has the potential to reduce zoonotic spillover risk, minimize antibiotic reliance, lessen environmental burdens, and obviate welfare harms inherent in conventional systems, aligning food system transformation with public health and sustainability objectives. This is because cultured meat production is considered an effective alternative to mitigate climate change and contribute to a more sustainable food system by reducing animal sacrifice. However, its development still faces significant challenges, especially those related to nutritional and regulatory aspects, which require further research and debate (Lee et al. 2022; Pakseresht et al. 2022; Post et al. 2020). Thus, this study compiles the most recent information on cultured meat through a scoping review, relating its aspects to food quality and safety.

### 1.1 Research Gaps and Novelty of the Present Review

Despite the rapid growth of scientific literature on cultured meat, existing review articles have tended to address the topic in a fragmented manner, often focusing on isolated dimensions such as technological development, environmental sustainability, ethical considerations, or consumer acceptance. Several reviews emphasize bioprocessing challenges, including cell sources, scaffolds, and bioreactor design, whereas others prioritize sustainability assessments, public perception, or ethical debates. However, relatively few studies integrate these dimensions within a single analytical framework that explicitly connects technological advances to food quality, nutritional performance, safety considerations, regulatory progress, and market readiness.

In particular, gaps remain regarding the systematic discussion of cultured meat from a food science and food safety perspective. Although technological feasibility has been extensively reviewed, less attention has been paid to how cell culture conditions, scaffolding materials, and media composition directly influence the nutritional quality, chemical and microbiological safety, and consistency of the final product. Similarly, although environmental and ethical benefits are frequently highlighted, these aspects are often discussed independently of regulatory requirements and consumer acceptance, limiting a holistic understanding of the barriers to large‐scale commercialization.

Another limitation of previous reviews is the lack of updated synthesis covering the most recent regulatory developments and commercial approvals, as well as emerging evidence on protein digestibility, lipid composition, and nutritional modulation strategies. Moreover, few reviews critically examine how regulatory heterogeneity across regions interacts with technological maturity and consumer perceptions, creating additional uncertainty for global market adoption.

In this context, the present review offers originality by adopting a multidisciplinary and integrated approach, synthesizing recent literature published between 2020 and 2025 to examine cultured meat through the combined lenses of technological development, nutritional aspects, environmental impact, ethical considerations, regulatory frameworks, and consumer acceptance. By explicitly linking production technologies to food quality, safety, and regulatory compliance, this review seeks to bridge existing gaps and provide a comprehensive perspective relevant to both researchers and stakeholders involved in the transition from laboratory‐scale innovation to market‐ready products.

### 1.2 Objectives of the Review

The objective of this scoping review is to compile and critically analyze recent scientific evidence on cultured meat, with an emphasis on its implications for food quality and safety. Specifically, this review aims to (i) summarize recent technological advances in cell sources, culture media, scaffolds, and bioprocessing strategies; (ii) evaluate the nutritional composition of cultured meat, with particular attention to protein and lipid content, digestibility, and opportunities for nutritional optimization; (iii) discuss environmental and ethical considerations associated with cultured meat production; (iv) examine current regulatory frameworks and approval pathways across different regions; and (v) analyze consumer acceptance trends and key factors influencing market adoption. Through this integrated analysis, the review seeks to identify persistent challenges, research gaps, and future directions necessary for the sustainable and safe commercialization of cultured meat.

## 2 Methodology

### 2.1 Search Strategy and Data Sources

The literature search on cultured meat focused on scientific articles published between 2020 and 2025. Data were collected from the ScienceDirect, Web of Science, and Scopus databases. The search was conducted between April 1, 2025, and August 31, 2025. The search was conducted using the following keywords: “Cultured meat OR lab‐grown meat” combined with the terms “AND production,” “AND nutritional components,” “AND sustainability,” “AND ethics,” “AND consumer acceptance,” “AND regulatory,” and “AND food safety.” In the search strategy, all keywords were cross‐referenced with the primary results to minimize the risk of missing relevant studies. Furthermore, to complement the automated search, the reference list of selected articles and review articles was also screened to identify additional relevant studies.

### 2.2 Eligibility Criteria

The inclusion criteria for articles on cultured meat were (i) use of keywords in their titles, abstracts, or keywords; (ii) being an original research article or a review article; (iii) published between 2020 and 2025; (iv) availability of complete data/information; and (v) published in English. On the other hand, short communications, letters, commentaries, and conference abstracts were excluded from the selection.

## 3 Development

### 3.1 Technological Development of Cultured Meat

The concept of cultured meat dates back nearly a century. As recently as the 1930s, Winston Churchill argued against raising whole animals when only specific parts were needed, proposing that these parts could be grown separately under suitable conditions (Graham 2025). Churchill proved to be remarkably visionary, as the first concrete advances in this area only began to appear more than 80 years later. Table 1 summarizes the main milestones in cultured meat development, from this initial conception to the most recent developments.

**TABLE 1:** Brief history of key events in cultured meat production.

Year	Event	References
2013	First beef cell‐based burger produced in the Netherlands	BBC News (2013)
2015	Startups emerge in the United States (Upside Foods) and Israel (SuperMeat)	SuperMeat (2025), UPSIDE Foods (2025)
2016	Nonprofit organization mercy for animals created the Good Food Institute (GFI), which works internationally to accelerate innovation in alternative proteins (including cultured meat)	GFI (Good Food Institute) (2025)
2019	First Brazilian startup (Ambi Realfood—Núcleo Vitro) to produce burgers from bovine cells	Stucchi (2021)
2020	Registration of approximately 60 cultured meat startups worldwide	Ellies‐Oury et al. (2022)
2021	The world's first cultured meat factory in Israel (Future Meat Technologies)	Euro Meat News (2021)
2021	Brazilian multinationals (JBS and BRF) invest in cultured meat	Constancio (2021), Souza (2021)
2022	Meatable from delft (the Netherlands) launches cultivated pork sausage	Sawers (2022)
2023	BioBetter announced that it had opened its first food‐grade pilot facility to accelerate the production of its growth factors for the cultured meat industry	Seleznyov (2023)
2024	Believer Meats (e.g., Future Meat Technologies)—EUA/Israel, has signed a strategic partnership agreement with GEA, one of the world's largest suppliers of production‐scale equipment and systems to the food, beverage, and pharmaceutical industries. The companies will focus on optimizing the performance, efficiency, and environmental impact of cultured meat production, starting with chicken and expanding to other products	Businesswire (2024)
2025	SuperMeat will develop new bioreactors that will allow for the production of whole cuts with greater realism and efficiency, optimizing muscle fibers and adipocytes	LLC (2025)
2025	Multus Biotechnology's announced the launch of its new food‐grade basal media, called DMEM/F12‐FG. The formulation is designed to deliver essential nutrients, including sugars, salts, minerals, and vitamins, that are critical for optimal cell growth in cultured meat production	Yates (2025)
2025	Vow (Australia/United States) operates the world's largest food‐grade cell culture bioreactor at 20,000 L	Mridul (2025)

This brief history demonstrates the rapid evolution of large‐scale cultured meat production. From the emergence of the first startups to technological improvements in bioreactors, culture media, and scalability, the sector is on a path to cost reduction. Investments are cautious but strategic, focusing on technology, regulation, and product differentiation (H. Gu, Kong, et al. 2025). However, the development and production of cultured meat raise critical questions, especially those related to ethical, technological, nutritional, and regulatory aspects, requiring further investigation and broader debate in the context of food quality and safety (Broucke et al. 2023).

**FIGURE 1:**
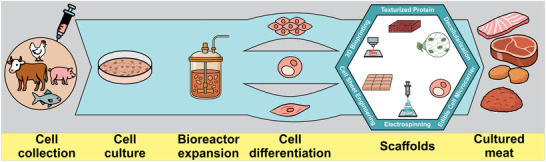
Schematic representation of the cultured meat production process. The main stages include cell sourcing through biopsy, expansion in culture medium, proliferation and differentiation in bioreactors, structuring with scaffolds, and the final processing into various meat products (e.g., ground meat, nuggets, and steaks).

Basically, the process of obtaining cultured meat can be summarized as follows (Post et al. 2020; Reiss et al. 2021; Ben‐Arye et al. 2020), as shown in Figure 1: (i) collection of a small fragment of tissue from a living animal by biopsy, performed under anesthesia. These cells are then isolated, dissected, and cultured in an artificial, nutrient‐rich environment containing amino acids, vitamins, and growth factors that stimulate their multiplication and differentiation into myotubes, the initial structures of muscle tissue; (ii) the cells are transferred to bioreactors, providing a controlled environment for temperature, pH, and oxygenation to simulate the physiological conditions of the animal body. In this phase, the cells proliferate and differentiate, forming muscle tissue on a larger scale; (iii) the last step involves organizing the tissues into scaffolds (Roy et al. 2021; Seah et al. 2022).

After all the steps involved in production, cultured meat can be applied in different forms, such as fresh meat (steaks, ground meat, chicken pieces) or as an ingredient in processed products, such as burgers, nuggets, meatballs, and sausages (Feddern et al. 2022). Depending on the final product, compounds such as flavorings, binders, and plant‐based additives or those obtained through fermentation can be incorporated, ensuring the necessary nutritional aspects (Ong et al. 2021). Additionally, the product may undergo additional treatments such as sterilization, pasteurization, smoking, fermentation, curing, or drying (Post et al. 2020).

To obtain cultured meat, four main inputs are required: the cell source, culture medium, bioreactors, and scaffold, which are three‐dimensional structures resembling a sponge, acting as a physical support for the cells to grow on them (Roy et al. 2021). One of the most critical challenges for the commercialization and large‐scale production of cultured meat lies in achieving robust cell expansion while preserving differentiation capacity and product quality. Although significant advances have been made in identifying suitable cell sources and developing edible scaffolds, cell expansion and differentiation remain biologically and economically constraining steps that directly influence scalability and cost competitiveness (Post et al. 2020; Lee et al. 2022).

The primary cell types employed in cultivated meat production include muscle satellite cells, mesenchymal stem cells, and fibro/adipogenic progenitors, which collectively represent the major cellular constituents of conventional meat tissue (Feddern et al. 2022). Among these, muscle satellite cells and other myogenic progenitors are widely considered the most suitable sources due to their inherent ability to proliferate and differentiate into myotubes. Nevertheless, their expansion capacity is biologically finite and highly sensitive to culture conditions, passage number, and biophysical stress, factors that become increasingly critical during large‐scale cultivation (Reiss et al. 2021; Takahashi et al. 2022). To address these constraints, recent research has focused on enhancing proliferative and differentiation potential while reducing dependence on recurrent animal biopsies, including the exploration of cell immortalization strategies to overcome natural limits on cell division (Giglio et al. 2024; Reiss et al. 2021). Despite these advances, repeated passaging during expansion remains associated with cellular senescence and phenotypic drift, which impair fusion efficiency and hinder the formation of mature muscle structures necessary for achieving desirable texture and protein quality in the final product (Guan et al. 2023).

Differentiation represents a second major bottleneck. Transitioning cells from a proliferative state to synchronized myogenic differentiation requires precise temporal modulation of biochemical cues and nutrient availability. Media formulations optimized for rapid proliferation often suppress myogenic signaling, whereas differentiation‐promoting conditions typically reduce biomass yield, creating a fundamental trade‐off between quantity and quality (Lee et al. 2022; X. Gu, Wang, et al. 2025). This challenge becomes increasingly pronounced during scale‐up, where heterogeneous microenvironments can lead to asynchronous differentiation and inconsistent tissue properties.

Scaling cell expansion from laboratory‐scale systems to industrial bioreactors introduces additional complexity. Suspension‐based cultures using microcarriers or edible cell carriers are among the most promising strategies to increase surface area and cell density, yet they expose cells to shear stress and mass transfer gradients that can negatively affect viability and differentiation potential (Norris et al. 2022; Yang et al. 2022). These effects are amplified at larger volumes, where precise control of oxygen, nutrients, and waste removal becomes increasingly difficult (Levi et al. 2022).

Importantly, scale‐up cannot be decoupled from downstream differentiation and structuring processes. Decisions regarding whether biomass is harvested as proliferative cells, partially differentiated tissues, or fully structured muscle constructs have direct implications for processing efficiency, product formulation, and sensory quality (Gurel et al. 2024; Lee et al. 2025). Consequently, scalable production requires integrated bioprocess designs that align cell expansion, differentiation, and structuring within economically viable operational frameworks.

Culture media represent one of the most critical components in cultivated meat production, as they directly govern cell proliferation, differentiation, and ultimately the characteristics of the final product. In parallel, discussions regarding the quality and safety of cultivated meat remain ongoing, with no universal consensus yet established. Nevertheless, the highly controlled conditions under which cell culture is performed have been shown to substantially reduce the risk of pathogen contamination, thereby offering potential food safety advantages over conventional meat production systems (X. Gu, Wang, et al. 2025).

Because cultivated meat is intended for human consumption, all inputs involved in the production process must comply with stringent food safety requirements. This includes not only culture media but also scaffold materials, which must be edible or generally recognized as safe (GRAS) to ensure consumer protection (Ong et al. 2021). However, many conventional scaffold manufacturing techniques—such as extrusion, decellularization, freeze‐drying, and electrospinning—are labor‐intensive and rely on specialized equipment and chemical treatments. These processes can increase the risk of contamination or residual chemical accumulation if not carefully controlled, underscoring the need for rigorous safety and quality standards throughout the production chain. Decellularization commonly relies on chemical detergents such as sodium dodecyl sulfate (SDS), Triton X‐100, or CHAPS to lyse cells and remove intracellular components; however, incomplete removal of these agents may result in residual cytotoxicity or chemical contamination of the scaffold (Ng and Kurisawa 2021; Levi et al. 2022). Electrospinning frequently requires organic solvents, including hexafluoro‐2‐propanol (HFIP), chloroform, dichloromethane, or trifluoroethanol, to dissolve polymers prior to fiber formation, raising concerns about solvent residues and occupational exposure if process control is insufficient (Seah et al. 2022; Fasciano et al. 2024). In contrast, freeze‐drying and extrusion are primarily physical processing techniques; however, they may still involve chemical cross‐linkers, plasticizers, or binders—such as glutaraldehyde, genipin, or calcium salts—depending on the scaffold formulation, which also require careful validation to ensure food safety (Nurul Alam et al. 2024; Levi et al. 2022).

From an economic standpoint, culture media remain the dominant cost driver in cultivated meat production and are among the least mature components from a commercialization perspective (Post et al. 2020; Feddern et al. 2022). The traditional reliance on fetal bovine serum (FBS) presents ethical, regulatory, and financial challenges, prompting extensive efforts to develop serum‐free and chemically defined media formulations (Lee et al. 2022). However, replacing serum without compromising cell proliferation and differentiation performance continues to be a major technical hurdle.

Recent studies have proposed several strategies to address media‐related bottlenecks. These include the production of recombinant growth factors through microbial or plant‐based expression systems, which aim to improve scalability and significantly reduce costs relative to pharmaceutical‐grade counterparts (Yates 2025; X. Gu, Wang, et al. 2025). In parallel, food‐grade basal media enriched with tailored amino acid compositions, carbohydrates, lipids, vitamins, and minerals are being designed to better align with the metabolic requirements of cells during both expansion and differentiation phases (Kim et al. 2023; Feddern et al. 2022).

Additional approaches involve the use of alternative bioactive supplements to partially substitute expensive growth factors. Plant‐derived protein hydrolysates and functional polysaccharides have shown potential to support cell adhesion, proliferation, and differentiation when incorporated into culture media or scaffold systems (Nurul Alam et al. 2024; Xiong et al. 2025). Despite their promise, these alternatives often exhibit cell‐type–dependent effects and require careful optimization to ensure reproducibility, consistency, and compliance with food safety regulations.

From a regulatory perspective, all culture media components must meet food‐grade standards and be assessed for residual presence in the final product, further constraining formulation strategies (FAO 2023a; Lanzoni et al. 2024). Consequently, media optimization extends beyond a purely biological challenge, emerging instead as a multidimensional issue that integrates cost reduction, regulatory acceptance, safety assurance, and process standardization—factors that are central to the successful commercialization of cultivated meat.

The combined challenges associated with cell expansion, differentiation control, and culture media formulation directly constrain the commercial viability of cultivated meat. High production costs—largely driven by media and bioprocess complexity—remain a major barrier to market competitiveness with conventional meat (Post et al. 2020; Caputo et al. 2024). Furthermore, variability in cellular behavior at scale raises concerns regarding product consistency, quality assurance, and regulatory approval.

Beyond technical constraints, successful commercialization depends on the ability to deliver products with acceptable sensory attributes, nutritional profiles, and safety assurances, all of which are intrinsically linked to effective control of expansion and differentiation processes (Broucke et al. 2023; Hadi and Brightwell 2021). Thus, advances in media optimization, scalable bioprocessing, and differentiation control are not incremental improvements but foundational requirements for transitioning cultivated meat from pilot‐scale demonstrations to industrial food systems.

#### 3.1.1 Scaffolds Requirements

**FIGURE 2:**
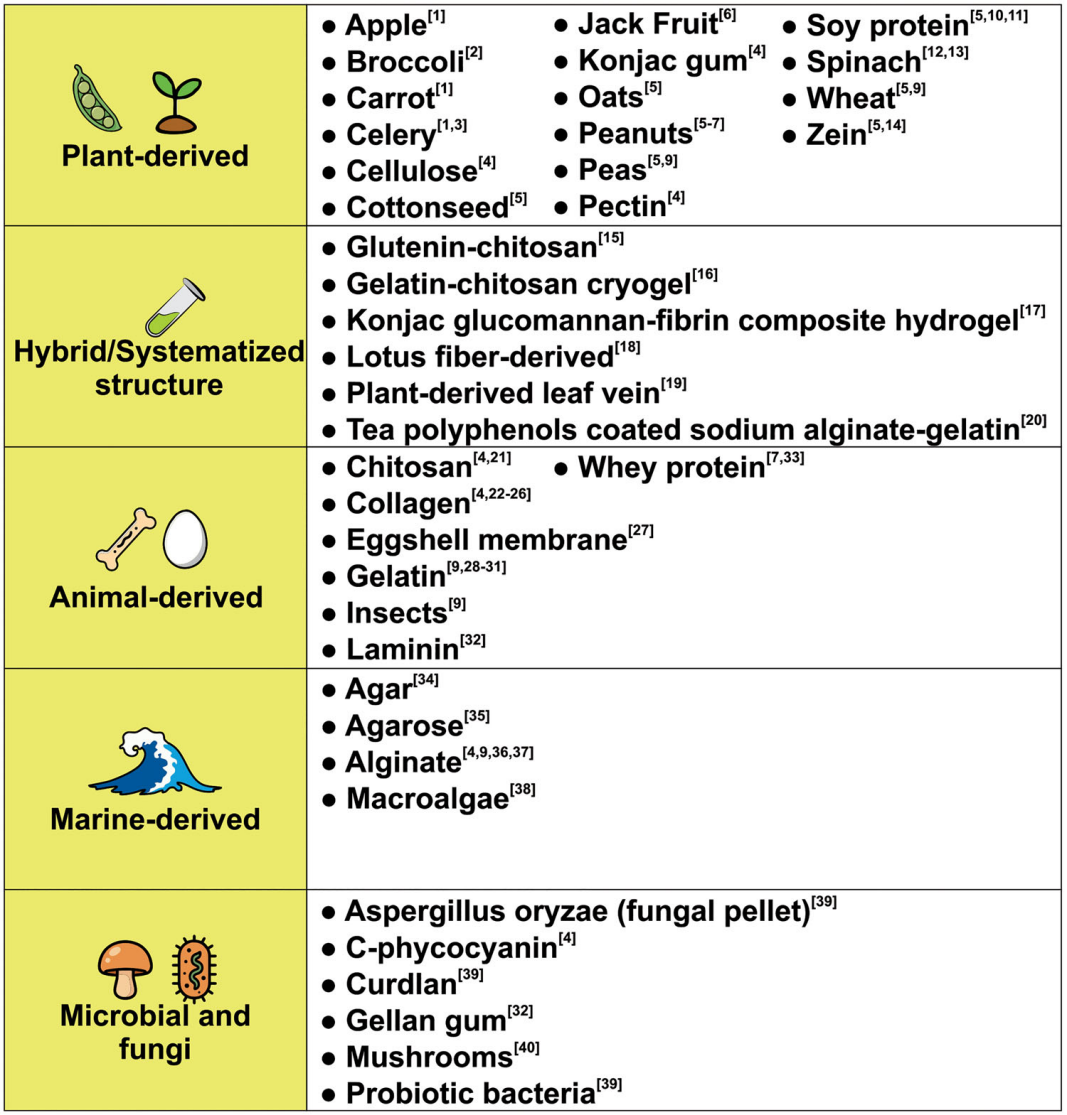
Materials investigated for scaffold development in cell culture. *Source*: Information extracted from [1] Contessi Negrini et al. (2020), [2] Thyden et al. (2022), [3] Campuzano et al. (2020), [4] Levi et al. (2022), [5] Cai et al. (2020), [6] Ong et al. (2021), [7] Post et al. (2020), [8] Zheng et al. (2022), [9] Dutta et al. (2022), [10] Ben‐Arye et al. (2020), [11] Geurs et al. (2025), [12] Jones et al. (2021), [13] Robbins et al. (2020), [14] Xiong et al. (2025), [15] Wu et al. (2024), [16] Kim et al. (2025), [17] Tang et al. (2024), [18] Wu et al. (2025), [19] Luo et al. (2024), [20] Chen et al. (2023), [21] Zernov et al. (2022), [22] Furuhashi et al. (2021), [23] Ng and Kurisawa (2021), [24] Takahashi et al. (2022), [25] Wang et al. (2023), [26] Yang et al. (2022), [27] Jang et al. (2024), [28] Chen et al. (2024), [29] M. Lee et al. (2022), [30] Norris et al. (2022), [31] Rao et al. (2023), [32] Alheib et al. (2022), [33] Pei et al. (2025), [34] Garcia‐Cruz et al. (2021), [35] You et al. (2025), [36] Li et al. (2021), [37] Lee et al. (2024), [38] Bar‐Shai et al. (2021), [39] Kolodkin‐Gal et al. (2024), [40] Yang et al. (2025).

Scaffolds are essential materials in the cultured meat production process, as they function as a template for tissue formation, mimicking the natural extracellular matrix so that cells can adhere and proliferate. Their porous network should facilitate the exchange of oxygen, nutrients, and waste elimination, promoting cellular metabolism and preventing necrosis. Scaffolds must possess desirable sensory properties, considering that they may be edible depending on the culture, which may also contribute nutritional value. Otherwise, they must be designed to be removable (Seah et al. 2022). Other necessary properties are stability, digestibility, textural characteristics, and water retention capacity (Nurul Alam et al. 2024). Furthermore, a differentiated structuring approach is required to create products that contain specific characteristics, such as a steak or a fillet, as well as differentiating cell types and arrangements to produce a familiar appearance and texture (Dutta et al. 2022). The main objective is to promote the development of muscle, adipose, and connective tissues, which can be processed later, using methods similar to those of traditional meat production, such as in hamburgers or sausages (Nurul Alam et al. 2024). This review further explores the use of different scaffolds, considering the role of food engineering in the cultured meat production process. Figure 2 exemplifies several studies that explore scaffolds from biological sources with the aim of reducing costs and minimizing the processing of natural materials.

Scaffolds can be fabricated from different classes of polymers, with protein‐based systems (e.g., fibrin, collagen, keratin, whey protein, and zein) representing one important category (Ben‐Arye et al. 2020; Furuhashi et al. 2021; Garcia‐Cruz et al. 2021; Guan et al. 2023; Ng and Kurisawa 2021; Norris et al. 2022; Pei et al. 2025; Rao et al. 2023; Takahashi et al. 2022; Xiong et al. 2025; Yang et al. 2022; Zernov et al. 2022), alongside polysaccharides and other biopolymers commonly explored for cultured meat applications. The proteins, especially those derived from plants, must possess appropriate mechanical characteristics, such as sufficient strength and toughness to support cell adhesion and proliferation without collapse, as well as elasticity and flexibility, allowing deformation without rupture and mimicking the natural texture of muscle tissue. It is also essential that they possess porosity and permeability, favoring the diffusion of nutrients, gases, and growth factors for proper cell development, and biocompatibility, ensuring compatibility with muscle cells, promoting their adhesion and proliferation without causing adverse responses (Nurul Alam et al. 2024; Samrot et al. 2023).

Despite the emphasis on animal welfare, some scaffolds still use animal byproducts such as skin, ligaments, intestines, and bones. However, this practice can contribute to the sustainability of traditional livestock farming by utilizing agro‐industrial waste; therefore, these types of scaffolds were not addressed in this review. Furthermore, as demonstrated in Figure 2, significant advances have been made in the development of animal‐free alternatives. Another important aspect in obtaining scaffolds is the need to minimize the use of nonedible and/or toxic compounds, including solvents and cross‐linkers, through strict control during the different stages of biomaterial processing, which are important aspects for ensuring the quality and safety of the final product (Nurul Alam et al. 2024).

Complex matrices have also been developed from plants and microorganisms such as lignins, decellularized leaves, algae, and bacterial and fungal mycelia (Bar‐Shai et al. 2021; Campuzano et al. 2020; Contessi Negrini et al. 2020; Jones et al. 2021; Kolodkin‐Gal et al. 2024; Luo et al. 2024; Robbins et al. 2020; Thyden et al. 2022; Wu et al. 2025). Polysaccharides, such as cellulose, starch, chitin, chitosan, agarose, and alginates, also emerge as promising materials for the production of scaffolds (Y. Chen, Li, et al. 2023; Dutta et al. 2022; Lee et al. 2024; Levi et al. 2022; Li et al. 2021; Wu et al. 2024; You et al. 2025; Zernov et al. 2022). These sources are often preferred as biomaterials due to their economic, sustainable, and ecological potential. On the basis of the above, Table 2 summarizes key advances in cultured meat production.

**TABLE 2:** Main aspects related to technological advances in obtaining cultured meat.

Aspects	Main results	References
Source of cells	Studies seek to reduce the need for biopsies	Lee et al. (2022), Post et al. (2020), Reiss et al. (2021)
Cell culture	Alternatives without fetal bovine serum are being explored	Levi et al. (2022), Nurul Alam et al. (2024), Seleznyov (2023), Yates (2025)
Bioreactors	Enabling large‐scale production	Businesswire (2024), Lee et al. (2022), LLC (2025), Post et al. (2020), UPSIDE Foods (2025), Yang et al. (2022)
Scaffolds	Edible or removable	Ben‐Arye et al. (2020), Furuhashi et al. (2021), Levi et al. (2022), Nurul Alam et al. (2024), Ong et al. (2021), Pei et al. (2025)

### 3.2 Nutritional Aspects

According to the USDA (United States Department of Agriculture) Food DataCentral (USDA 2024), 100 g of cooked beef has the following nutritional composition: 25.93 g of protein, 15.13 g of total lipids, 0 g of carbohydrates, 2.15 mg of iron, 5.84 mg of zinc, 84 mg of cholesterol, 72 mg of sodium, and 57.47 g of water. The lipid content can vary depending on the cut of meat, directly influencing the ratio of saturated to unsaturated fatty acids. Proteins derived from animals are considered complete, as they contain all the essential amino acids. Essential amino acids are those that the body cannot produce and must obtain through diet, such as leucine, lysine, and tryptophan. Nonessential amino acids, such as alanine, glutamine, and glycine, can be synthesized by the body. Furthermore, animal proteins have high bioavailability due to their easy digestibility (Gurel et al. 2024). Therefore, producing a nutritionally valuable product in the laboratory involves several challenges, mainly those related to the physicochemical composition.

Y. Chen, Pius Bassey, et al. (2023) demonstrated that muscle stem cells derived from male piglets exhibited increased protein expression when cultured in a hydrogel. However, when the amino acid profile of cultured meat was compared with pork, it was found that the amino acid content of cultured meat was lower (11.24%) than that of pork (21.89%). Kim et al. (2023) reported that chicken muscle tissues cultured at an optimal temperature of 41°C increased the amino acid yield in cultured chicken muscle tissue (4.8 g/100 g), thereby improving the nutritional value of cultured meat. However, the amino acid content in cultured chicken muscles remained lower than that of conventional chicken meat (22 g/100 g). Tanaka et al. (2022) demonstrated the “cell sheet‐based meat,” a scaffold‐free approach using cell sheet technology, and characterized its texture and nutrients. The wet weight percentage of total protein in the cell sheet (6.3) was about half that of beef (11.3). On the other hand, the dry weight percentage of total protein in the bovine myoblast cell sheets in the study was 50.6, compared to 28.8 in beef, significantly lower than that of the bovine myoblast cell sheet. This difference is due to the high‐water content of the bovine myoblast cell sheets. Zhu et al. (2022) demonstrated that treatment of porcine muscle stem cells with l‐ascorbic acid 2‐phosphate increased the production of several amino acids, such as glutamic acid, glycine, arginine, proline, alanine, leucine, tyrosine, phenylalanine, lysine, aspartic acid, threonine, serine, valine, and histidine, by 1.2‐ to 1.5‐fold relative to the control sample. However, these results are still inferior to those of traditional meat.

These findings highlight that, although significant progress has been made in enhancing the nutritional profile of cultured meat through different strategies—such as optimization of culture conditions, scaffold technologies, and supplementation with bioactive compounds—the amino acid and protein content of cultured meat still falls short of conventional meat. This reinforces the need for further research aimed at refining culture systems and identifying innovative approaches to bridge this nutritional gap, ensuring that cultured meat can not only mimic but also potentially match or surpass the quality of traditional animal‐derived products.

To date, limited studies have directly compared the protein digestibility of cultured meat and conventional meat. Metabolomic analyses comparing cultured and traditional chicken meat have shown overall nutritional similarity, while also revealing differences in metabolites associated with protein and amino acid metabolism, suggesting that digestive behavior may not be identical between the two products (Park et al. 2025). In parallel, critical reviews have highlighted the lack of standardized nutritional evaluations, emphasizing the need for targeted studies assessing protein digestibility and bioavailability of cultured meat using established methods (Nutra Horizons 2024). Addressing this gap, recent experimental evidence demonstrated that proteins derived from cultured meat exhibit significantly higher in vitro digestibility and generate a greater number of potential bioactive peptides compared to proteins from conventional animal meat and plant‐based sources (Xie et al. 2026). Together, these findings indicate that although comprehensive in vivo data are still limited, emerging evidence suggests that cultured meat proteins may display favorable digestibility characteristics, warranting further investigation within the context of food quality and safety.

In addition to proteins, lipids are indispensable components of the human diet, contributing to the flavor, juiciness, and tenderness of meat (Gurel et al. 2024). The fat content of cultured meat is not intrinsic and may vary widely depending on the production strategy. In many current prototypes, the absence or limited incorporation of adipose tissue results in lower total lipid levels, which may negatively affect sensory attributes (Post et al. 2020; Broucke et al. 2023). However, both lipid quantity and fatty acid composition can be intentionally modulated through the inclusion of cultured adipocytes and tailored culture conditions, enabling the reduction of saturated fatty acids compared to conventional meat (Feddern et al. 2022; Nutra Horizons 2024; Louis et al. 2023; Lee et al. 2025). Despite this nutritional flexibility, comprehensive and standardized data on the exact lipid content and lipid composition of final cultured meat products remain limited (Broucke et al. 2023; Park et al. 2025).

Xu et al. (2023) developed cultured fish fillets using a 1.7:1 muscle:fat ratio; the native tissue mimetic fiber array took 17 days to develop, resulting in a textural quality similar to that of natural meat. Tsuruwaka and Shimada (2022) used fins obtained from seafood waste to produce cultured meat. Cell culture resulted in adipocyte‐like cells characterized by white droplets, confirming the presence of white adipocytes, as assessed by fatty acid‐binding protein 4 and adiponectin using immunofluorescence and real‐time polymerase chain reaction techniques. Sample characterization indicated the presence of myristic acid, pentadecanoic acid, palmitic acid, palmitoleic acid, octadecanoic acid, oleic acid, linoleic acid, arachidonic acid, and docosahexaenoic acid, whose fatty acid profile is similar to that typically found in seafood.

Song et al. (2022) used porcine adipose progenitor cells in comparison with porcine subcutaneous adipose tissues; the results demonstrated that porcine adipose progenitor cells exhibited similarity with the fatty acid profile of porcine subcutaneous adipose tissue in terms of typical pork flavor, including linoleic acid, oleic acid, and nonadecenoic acid. Louis et al. (2023) used bovine muscle adipose tissue and conducted studies with two culture media: Dulbecco's Modified Eagle Medium (DMEM) and IntegriCulture Minimum Essential Media (IMEM), supplemented with 100 µmol/L of different free fatty acids: phytanic, pristanic, oleic, palmitoleic, myristoleic, and elaidic. IMEM showed a slightly higher fatty acid composition, with a 57‐fold increase in cell number compared to a 53‐fold increase in DMEM. The results demonstrated that cultured meat, when compared with beef tallow and intramuscular fat, exhibited a similar fatty acid composition. This comparison demonstrates that cultured fat could replicate the sensory characteristics of conventional beef fat.

Kang et al. (2021) developed a technology using Wagyu bovine satellite cells to construct whole‐meat tissue, including muscle fibers, fat, and blood vessel cells. These cells were encapsulated or used from stem cells derived from bovine adipose tissue. The fibers were applied to scaffolds that mimic tendons, helping to maintain fiber structure and cell alignment. The results indicated that it was possible to produce meat‐like tissue, forming a structured tissue 5 mm in diameter and 10 mm in length, resembling a real Wagyu beef steak.

In addition to reproducing the nutritional composition of conventional meat, cultured meat represents an alternative that allows for nutritional improvements, such as increased iron content and essential unsaturated fatty acids (omega‐3, omega‐6, and omega‐9) (Caputo et al. 2024). However, there are still obstacles to be overcome, especially regarding the addition of certain nutrients found in traditional meats, such as vitamins and minerals, without compromising stability or increasing rancidity (e.g., Chriki and Hocquette 2020). However, products already commercially available, such as Good Meat's cultured chicken, have nutritional values similar to conventional chicken: 180 calories per 100 g, 16 g of protein, 3.3 g of fat, and 1.1 mg of iron (Good Meat 2025).

Collectively, these studies demonstrate that cultured meat and fat can closely mimic the fatty acid composition, texture, and nutritional properties of conventional animal products, while also offering opportunities to enhance certain nutrients (Post et al. 2020; Broucke et al. 2023; Louis et al. 2023; Giglio et al. 2024). Continued innovation in cell culture techniques, scaffold design, and nutrient fortification will be essential to produce cultured meat that is not only comparable to but potentially superior in nutritional quality, safety, and sustainability (Post et al. 2020; Levi et al. 2022; Fasciano et al. 2024; X. Gu, Wang, et al. 2025).

Despite advances, the nutritional composition of cultured meat and its derivatives, particularly protein content, still requires improvement to meet the nutritional demands of consumers (Chriki and Hocquette 2020; Broucke et al. 2023). Most current studies focus on optimizing growth media to produce nutrient‐rich cells, demonstrating the possibility of adjusting nutritional composition by modifying culture medium conditions (Lee et al. 2022; X. Gu, Wang, et al. 2025; H. Gu, Kong, et al. 2025). Therefore, production technologies and innovation associated with cultured meat represent not only a nutritional alternative but also a continuous advance in the search for sustainable and ethical food practices compared to conventional animal protein production (Post et al. 2020; Feddern et al. 2022; H. Gu, Kong, et al. 2025).

### 3.3 Environmental Issues

Cultured meat represents an alternative to conventional meat, capable of addressing the growing global demand for protein while reducing the environmental and ethical impacts associated with conventional livestock production. Conventional meat production is considered inefficient, as these organisms consume large quantities of food, with much of the energy being directed toward sustaining their metabolism and forming tissues that are not consumed, including bones, tendons, and hides. In contrast, cultured meat consists exclusively of edible structures (Feddern et al. 2022; Good Food Institute 2025). For this alternative to become viable on a large scale, it is essential that production be economically viable, considering the differences in manufacturing processes for products like ground beef and whole cuts. The final stage of production, which involves the formation of muscle tissue, also requires optimized strategies to maximize its efficiency (Dutta et al. 2022). One of the main sustainability challenges in cultured meat production lies in the cell culture medium, which uses animal‐derived components. Therefore, alternatives to these media have been developed to reduce environmental impact and align production with the United Nations Sustainable Development Goals (SDGs) (Post et al. 2020; UN (United Nations) 2015).

Compared to conventional livestock farming, cultured meat production consumes fewer natural resources, such as land and water, and has a reduced carbon footprint—a measure of the total amount of GHGs, primarily carbon dioxide (CO_2_), emitted directly or indirectly throughout its life cycle, helping to alleviate pressure on the environment (Hadi and Brightwell 2021; IPCC (Painel Intragovernamental de Mudanças Climáticas) 2018).

Although global demand for meat continues to grow, traditional livestock production remains a major source of GHG emissions, many of which have a warming potential greater than that of CO_2_ (Nugrahaeningtyas et al. 2024). According to an FAO report, cattle are the main contributors to livestock GHG emissions, responsible for about 3.8 Gt CO_2_ eq per year (62%). Other species, including pigs, chickens, buffaloes, and small ruminants, account for smaller shares (14%, 9%, 8%, and 7%, respectively). Globally, direct emissions (mainly CH_4_ from enteric fermentation and manure management, plus N_2_O) reach 3.7 Gt CO_2_ eq (∼60%), whereas indirect emissions from feed and input production contribute 40% (2.6 Gt CO_2_ eq). Methane is the dominant gas, accounting for 54% of emissions, followed by CO_2_ (31%) and N_2_O (15%) (FAO 2023d).

**FIGURE 3:**
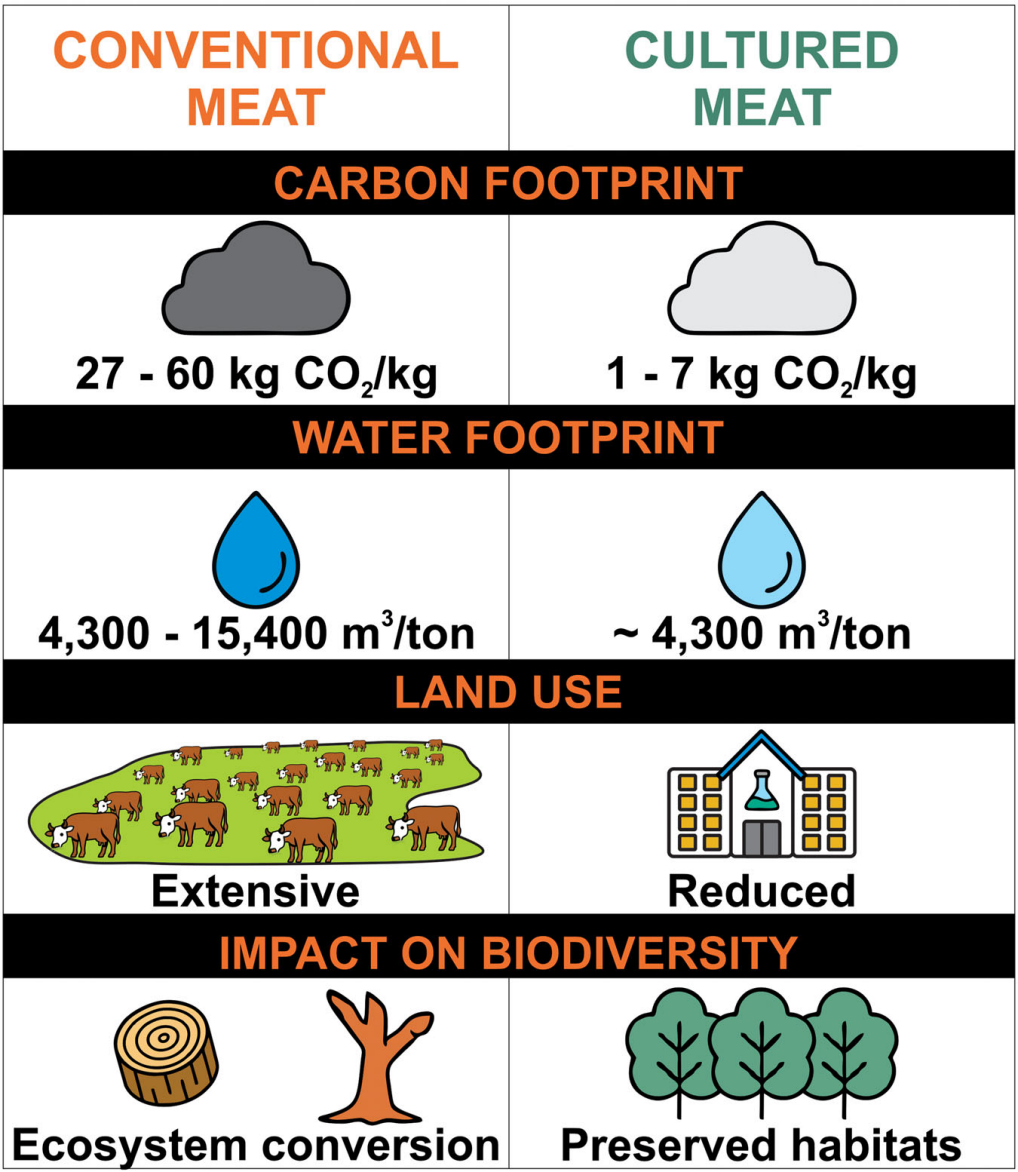
Comparative analysis of environmental impacts between conventional and cultured meat. Indicators include carbon footprint (kg CO_2_ eq/kg), water footprint (m^3^/ton), and land use. Cultured meat demonstrates significant reductions in greenhouse gas emissions, water consumption, and land occupation. *Source*: Information extracted from Dupont et al. (2022), Hadi and Brightwell (2021), Lee et al. (2025), Munteanu et al. (2021), Rodríguez Escobar et al. (2021).

These statistics reinforce the need to assess the carbon footprint associated with lab‐grown foods. Even though cultured meat has the potential to present a smaller carbon footprint than conventional beef, particularly under optimized production scenarios, it is crucial to evaluate the energy consumption involved in its production process, as energy demand remains one of the main determinants of its overall environmental performance (Post et al. 2020; H. Gu, Kong, et al. 2025; Good Food Institute 2025). This includes the planning and creation of advanced and responsive bioreactors capable of optimizing muscle cell growth and differentiation more efficiently than traditional bioreactors, saving time. Figure 3 presents a comparative analysis of environmental aspects, including the carbon footprint, of conventional versus cultured meat. The values presented are estimates, considering the most prevalent factors that affect the carbon footprint of cultured meat production, such as the culture medium and bioreactors, scaffolding, production scale, and vehicle transportation.

Although Figure 3 highlights the potential environmental advantages of cultured meat, it is important to acknowledge that these benefits remain largely based on projections and life cycle assessments rather than large‐scale industrial evidence. Although reductions in GHG emissions, water use, land occupation, and biodiversity impacts are promising, uncertainties persist regarding the scalability of production, energy demand of bioreactors, and the sourcing of growth media (Post et al. 2020; Broucke et al. 2023; Lee et al. 2022; H. Gu, Kong, et al. 2025; FAO 2023a; Good Food Institute 2025; Nutra Horizons 2024; X. Gu, Wang, et al. 2025). Moreover, the environmental performance of cultured meat will depend heavily on future technological innovations and the transition toward renewable energy systems. Thus, although cultured meat shows considerable potential to mitigate some of the environmental burdens of conventional livestock production, further empirical research and industrial data are required to validate its long‐term sustainability.

### 3.4 Ethical Considerations

In the ethical field, cultured meat also raises relevant discussions. On one hand, its development promises to eliminate animal suffering by avoiding farming and slaughter. On the other hand, it still uses animals as a source of cells, which raises questions about the true scope of animal welfare in this model (Anomaly et al. 2024). It can be considered that the production of cultured meat is based on ethical aspects in relation to the use of animals for human consumption, as the meat industry in general (poultry, cattle, fish, or pigs) presents critical breeding conditions, such as overconfinement and mistreatment, and requires animal slaughter to obtain the final product. Anomaly et al. (2024) highlight the immense animal suffering on factory farms and the public health consequences associated with intensive practices, emphasizing that cultured meat can contribute to mitigating these threats, including in light of pandemics such as COVID‐19, which raised awareness of the potential dangers of zoonotic diseases. Other authors argue that organizations dedicated to defending animal welfare and public health should support research and development to make cultured meat significantly cheaper and more accessible.

However, ethical concerns related to animal use are not uniform across species, and their relevance may vary depending on biological, behavioral, and production‐system characteristics. Ethical assessments increasingly consider factors such as animal sentience, cognitive complexity, and the capacity to experience pain and distress—criteria now being reflected in contemporary legal frameworks recognizing animal sentience (UK Public General Acts 2022). For terrestrial livestock such as poultry and cattle, intensive confinement and routine slaughter practices are widely criticized for generating significant welfare deficits (Hadi and Brightwell 2021; Santana et al. 2025). In contrast, the ethical implications of seafood production are more heterogeneous, and academic attention to ethics in cultured fish is only emerging, with scholars noting distinct normative challenges when comparing aquatic species to more traditionally farmed animals (Ferrari 2025; Mullan et al. 2024). These differences in sentient capacity, societal attitudes, and production contexts contribute to variability in moral concern across species categories, reinforcing the need for nuanced ethical evaluation rather than a one‐size‐fits‐all approach.

Another key ethical and safety concern in cultured meat production is the composition of culture media, particularly the historical reliance on animal‐derived components such as FBS. Beyond ethical objections related to animal welfare and slaughter, the use of FBS raises significant microbiological and chemical safety concerns due to its biological variability, lack of full compositional definition, and susceptibility to contamination. These characteristics complicate hazard identification, risk assessment, and traceability, which are central principles in food safety governance and ethical responsibility toward consumers (FAO 2023a; Hadi and Brightwell 2021; Lee et al. 2022).

From a microbiological standpoint, culture media are intrinsically high‐risk matrices, as they are rich in nutrients, growth factors, and bioavailable carbon and nitrogen sources that can support the rapid proliferation of bacteria, fungi, yeasts, and mycoplasma if contamination occurs. Although cultured meat production is performed under controlled conditions, the extended duration of cell cultivation, repeated handling steps, and scale‐up to industrial bioreactors increase the probability of contamination events, particularly in open or semi‐open systems (Post et al. 2020; Broucke et al. 2023; FAO 2023a). Ethically, the potential introduction of pathogenic or spoilage microorganisms challenges the claim that cultured meat inherently offers superior public health outcomes, emphasizing the obligation to demonstrate safety advantages rather than assume them (Anomaly et al. 2024).

Chemical safety concerns associated with culture media further reinforce the ethical dimension of cultured meat production. Media formulations may include antibiotics, antimycotics, recombinant growth factors, hormones, buffers, and other bioactive compounds intended to maintain cell viability and productivity. Residual presence of these substances, as well as their degradation products, in the final product raises toxicological uncertainties and long‐term exposure concerns, particularly given the absence of extensive consumption history for cell‐based foods (FAO 2023a; Hadi and Brightwell 2021; Lanzoni et al. 2024). From an ethical perspective, these uncertainties relate to the principles of precaution and informed consumer choice, as the introduction of foods with poorly characterized chemical residues may undermine consumer trust and autonomy (Broucke et al. 2023).

The use of antibiotics in culture media also presents broader ethical and societal implications, particularly in the context of global efforts to mitigate antimicrobial resistance. Although antibiotics are employed to prevent microbial contamination rather than treat disease, their routine or preventive use contradicts emerging regulatory and ethical frameworks that promote antibiotic‐free food production systems (FAO 2023a; Hadi and Brightwell 2021). Consequently, the reduction or elimination of antimicrobial agents in culture media is not only a technical challenge but also an ethical imperative aligned with public health objectives.

In response to these microbiological, chemical, and ethical challenges, significant efforts have been directed toward the development of serum‐free and chemically defined culture media. Such media reduce biological variability, facilitate hazard identification, and improve transparency regarding input materials, thereby supporting both regulatory oversight and ethical accountability (Lee et al. 2022; X. Gu, Wang, et al. 2025). In parallel, the implementation of closed, automated, and monitored production systems, combined with aseptic processing, environmental monitoring, and hazard analysis approaches analogous to HACCP, is increasingly recognized as essential to minimize contamination risks across the entire production chain (Post et al. 2020; FAO 2023a; Lanzoni et al. 2024).

Collectively, ensuring the microbiological and chemical safety of culture media is not merely a technical requirement but a foundational ethical obligation in cultured meat production. Transparent control of inputs, robust risk management, and alignment with public health and sustainability goals are critical to substantiate ethical claims regarding consumer safety, environmental responsibility, and the societal benefits of this emerging food technology (Anomaly et al. 2024; FAO 2023a).

The use of animal‐derived biomaterials is also used to create scaffolds for cell culture. Although biomaterials, such as collagen and alginate, provide an environment that simulates the conditions of natural meat, their use contradicts the fundamental goal of cultured meat, which is to minimize environmental impacts and reduce production costs. The need to keep and sacrifice animals to obtain these materials not only perpetuates animal suffering while also resulting in high costs, as these biomaterials are expensive and their properties vary from animal to animal. Furthermore, the lack of reproducibility of these biomaterials, due to natural variability among individuals of the same species, raises concerns about the sustainability and efficiency of cultured meat, challenging the ethical rationale for using animal resources in this context (Fasciano et al. 2024). Montefiore and Goris (2024) report that the debate surrounding cultured meat presents a complex ethical dilemma, highlighting the difficulty of consistently addressing its acceptance or rejection.

### 3.5 Regulatory Aspects

Table 3 provides an overview of regulatory developments of cultured meat in different parts of the world.

**TABLE 3:** Global regulatory landscape of cultured meat as of 2025.

**Country/Region**	**Regulatory agency/legal basis**	**Description**	**Status**	**Are cultured meat products sold commercially?**	**Reference**
Singapore	Singapore Food Agency (SFA) Framework de Novel Food	The first country to authorize the sale of cultured meat in 2020, following the authorization granted to the food tech company Eat Just to market cultured chicken meat	Approved	Yes	SFA (2025)
United States of America—USA (Federal)	FDA (pre‐market), USDA–FSIS (labeling and inspection)	Second country to approve the sale of cultured chicken meat, in 2023, by Good Meat	Approved	Yes	FDA (2025)
USA—Florida	State law, SB 1084 (2024)	Prohibits the manufacture/sale/distribution of cultured meat (effective July 2024)	Prohibition	No	Florida Senate (2024)
USA—Alabama	State law, SB 23 (2024)	Prohibits the manufacture/sale/distribution of cultured meat. Classified as a misdemeanor (Class C misdemeanor) starting October 2024	Prohibition	No	Hess (2024)
Israel	Ministry of Health	Strong research and development activity and regulatory discussions. In 2023, the country became the first to approve the sale of beef cultivated through Aleph Farms	Approved	Yes	Ministry of Health (2024)
European Union	EFSA (EU Regulation) 2015/2283—Novel Foods Regulation	EFSA authorization required; no approval until September 2025	Under analysis	No	EC (2025), EU (2025)
Italy	National law (2023)	National ban on the production and sale of cultured meat	Prohibition	No	Amante (2023)
Australia/New Zealand	FSANZ—Food Standards Code—Standard 1.5.1/Schedule 25	Cultured Quail Meat Approval (Vow, A1269) in 2025	Approved	Yes	FSANZ (Food Standards Australia New Zealand) (2025)
Brazil	ANVISA (novel foods) and MAPA (regulation) (RDC No. 839)	Regulation on the registration of new foods and ingredients, including those derived from cell culture, such as cultured meat. No approval until September 2025	Under analysis	No	Brasil Anvisa (2023)

*Note*: Countries highlighted in green represent those with approved commercialization (Singapore, United States, Israel, Australia/New Zealand); yellow indicates regions with ongoing regulatory assessment (Brazil, European Union); and blue indicates prohibition (Italy, Florida, Alabama).

Singapore was the first country to approve the commercial sale of cultured meat. This requires pre‐market approval, involving the submission of safety assessments to the Singapore Food Agency. These assessments are conducted through specific documentation that assesses the safety of substances used during the production or manufacturing of novel foods, serving as the basis for commercial approval. Although there is no specific protocol, the agency expects production to comply with good laboratory practices (FAO 2023a).

The United States became the second country to approve the commercialization of cultured chicken meat, in 2023, by the company Good Meat. This was despite the lack of specific legislation for novel foods, relying instead on interagency agreements, including the Food and Drug Administration (FDA) and the USDA through the Food Safety and Inspection Service (FSIS). The FDA is responsible for overseeing the initial stages of the process, such as cell collection, cell banking, and cell growth, whereas the USDA–FSIS regulates the production, labeling, and inspection of products considered “meat” and “poultry products” under the Federal Meat Inspection Act (FMIA) and the Poultry Products Inspection Act (PPIA). The USDA also conducts inspections of production facilities and requires companies to submit samples for testing. In June 2022, this regulatory framework was expanded to include products derived from poultry cells. New labeling guidance was released, requiring companies to use specific terms such as “cell‐cultured” or “cultured” (FDA 2025).

Israel has also made significant progress in this sector. In January 2024, the Ministry of Health's National Food Service, responsible for ensuring the safety, quality, and authenticity of food for consumers, granted regulatory approval for Aleph Farms to market its products in the country. The country is the first to allow the sale of cultured beef (Grosglik et al. 2024). Products must undergo safety assessments, comply with labeling requirements, and obtain a license from the Ministry of Health. In September 2022, Israel announced a pilot program to test the safety and efficacy of cultured products, involving testing different types of meat products and monitoring consumer perceptions. Israel is home to innovative companies such as Aleph Farms, which developed the first cell‐based steak in 2018, and Meat‐Tech 3D, the first publicly listed cultured meat company (Ministry of Health 2024).

In 2018, the European Union (EU) established a regulatory framework for novel foods—including cultured meat. According to the regulation, any food that was not consumed to a significant extent in the EU before May 1997 is considered a novel food and must undergo a safety assessment before being marketed. The European Food Safety Authority (EFSA) is responsible for this assessment. Therefore, no cultured meat product can be marketed in the EU without EFSA authorization. Subsequent approval is required from the European Commission, which is responsible for risk management, which can be influenced by political and ethical issues. The main applicable legislation is the Novel Foods Regulation and, in some cases, legislation on genetically modified organisms (GMOs). The application of each regulation depends on the cells used in the cultivation: For example, foods derived from induced pluripotent stem cells (iPSCs) fall under GMO legislation, whereas other cases follow the Novel Foods Regulation. EFSA states that cultured meat and its derivatives must undergo rigorous safety assessment processes. This process includes the submission of a technical dossier demonstrating the product's safety. After the dossier is approved, the food will be included on the Union's list of novel foods, allowing other operators to market it without further authorization (EC 2025).

Some European countries still show resistance to cultured meat, such as Italy, whose government proposed a law in March 2023 prohibiting the production, use, and commercialization of food and feed made from cells or tissues derived from animals, reflecting the complexity of the conflicts between food safety, cultural values, ethics, and political interests (Formici 2023). In July 2022, the European Parliament passed a resolution calling on the European Commission to establish a legal framework for the production and labeling of cultured meat, emphasizing the need for clear labeling to ensure consumers can make informed choices. This case illustrates the interplay between food safety, cultural values, and policy in regulating innovative foods (Lanzoni et al. 2024).

On June 18, 2025, amendments to the Food Standards Code of Australia New Zealand (FSANZ) were gazetted, officially authorizing cell‐cultured quail for human consumption, following an application submitted by Vow Group Pty Ltd. To support this approval and future developments in the sector, FSANZ introduced a comprehensive regulatory framework that establishes mandatory labeling with the terms “cell‐cultured” or “cell‐cultivated”; sets food safety requirements for production and processing; lists approved cell‐cultured foods, starting with quail; and specifies microbiological limits for pathogens such as *Salmonella* spp. and *Listeria monocytogenes*. Importantly, all cell‐cultured products remain subject to pre‐market safety assessments, ensuring public health protection while providing regulatory clarity, fostering international alignment, and enabling informed consumer choice (FSANZ (Food Standards Australia New Zealand) 2025).

In Brazil, the National Health Surveillance Agency (ANVISA) published on December 18, 2023, Resolution RDC No. 839, which regulates the registration of new foods and ingredients, including those derived from cell culture, such as cultured meat, in addition to presenting the requirements for proving the safety and authorization of use of cultured meat and its derivatives (Brasil Anvisa 2023).

In other countries, the regulatory landscape for cell‐cultured meat remains at an early stage, with frameworks under development but no commercial authorizations to date. In the United Kingdom, the Food Standards Agency (FSA) applies its post‐Brexit novel foods regime, largely aligned with the EU model, and is currently revising procedures to streamline pre‐market evaluations (FSA‐UK (Food Standards Agency—United Kingdom) 2025). Japan, through the Ministry of Health, Labour and Welfare (MHLW) and the Ministry of Agriculture, Forestry and Fisheries (MAFF), has initiated working groups to establish safety and labeling standards for “cell‐cultured foods,” though no approvals have yet been granted (JACA (Japan Association of Cellular Agriculture) 2024). Similarly, the Ministry of Food and Drug Safety (MFDS) in South Korea is preparing technical guidance for safety assessment and market entry of cultured meat products, but no applications have reached commercialization (Mridul 2024). In China, the National Health Commission (NHC) and the State Administration for Market Regulation (SAMR) regulate such products under the novel food approval framework, which requires extensive safety dossiers; however, public approvals have not been announced (NHC (National Health Commission of the People's Republic of China) 2025). Canada regulates cell‐cultured meat as a “novel food” under the Food and Drugs Act, with Health Canada clarifying that commercialization requires a full pre‐market safety assessment; as of 2025, no cultured meat products have been authorized (Health Canada 2022).

Overall, the regulatory landscape for cultured meat remains highly heterogeneous: some regions have authorized commercial sales, while others remain in exploratory or regulatory design phases. This uneven progression highlights both the complexity of integrating novel food technologies into existing frameworks and the importance of harmonized, science‐based approaches ensuring safety, transparency, and access to global markets.

### 3.6 Consumer Acceptance

One of the main challenges for consumer acceptance cultured meat is achieving flavor and texture comparable to conventional meat, as flavor is one of the most determining factors for consumption and consumer loyalty (Nurul Alam et al. 2024). Although many consumers express interest in consuming cultured meat and its derivatives, studies consistently indicate that willingness to pay a premium price is substantially lower, with only around half of respondents—or fewer, depending on the market and study design—reporting acceptance of higher prices compared to conventional meat (Pakseresht et al. 2022; Bekker et al. 2021; de Oliveira Padilha et al. 2022; B. Chen, Zhou, et al. 2023; Wang et al. 2024). Therefore, reducing the production costs of biomaterial scaffolds is essential for commercial viability (Dutta et al. 2022). Another significant obstacle concerns nomenclature and categorization. Uncertainties remain regarding its classification—should it be considered actual meat or a meat substitute? The study by Bekker et al. (2021), with a sample of 482 respondents, revealed that participants were unable to clearly classify the product into any of these categories, highlighting the importance of labeling in this discussion. In light of these acceptance challenges, Table 4 summarizes key research findings on consumer perceptions of cultured meat, as well as purchase intentions.

**TABLE 4:** Summary of key findings from consumer research on the acceptability of cultured meat.

Number of respondents (*n*)	Aim	Results	Reference
1078	Check Australians’ beliefs about conventional meat, plant‐based meat, and cultured meat	Beliefs related to plant‐based meats were more positive than those related to cultured meat. 25% of participants indicated a willingness to consume cultured chicken and beef	de Oliveira Padilha et al. (2022)
4841	To evaluate the acceptance of cultured meat in different provinces of China, based on the relationship between food technophobia, sensitivity to food disgust, and acceptance of cultured meat	The level of acceptance was considered moderately low. Men and individuals considered healthy were more likely to accept cultured meat	Sheng et al. (2025)
1427	To investigate consumer acceptance of cultured meat in Belgium, Chile, and China, countries with distinct meat consumption patterns and cultural contexts	In general, cultured meat was perceived as animal‐friendly and innovative. Consumers from China emerged as the most open to consuming cultured meat, followed by those from Chile and Belgium. Differences in acceptance may relate to how meat attachment affects the perceived wholesomeness of cultured meat across countries	Rodríguez Escobar et al. (2025)
1224	Analyze the acceptance of the Asian population towards plant‐based meat alternatives, cultured meat, and insect‐based products	Intention to consume plant‐based meat alternatives was highest, followed by cultured meat and insect‐based products. The perception of unnaturalness was the strongest barrier to consumption intention, and this perception was strongest for cultured meat. Men and those more familiar with the products were more willing to consume alternative protein foods. Participants were willing to pay more for alternative protein foods if they were concerned about drug residues in the meat	Chia et al. (2024)
1105	Explore preferences between plant‐based products, hybrids (plant‐based and conventional meat), and cultured meat in Belgian	Plant‐based meat analogue products had the highest acceptance, and cultured meat products had the lowest acceptance	Coucke et al. (2023)
1180	Assess Chinese consumers’ willingness to pay (WTP) for plant‐based meat and cultured meat; specifically, examine the effects of six different types of information on consumers’ WTPs	Positive/Negative information has positive/negative effects on consumers’ WTP. With positive information, both nutritional and environmental information increase consumers’ WTPs. Specific information has a larger effect than general information, only for positive nutritional information. Knowledge about these meat alternatives, self‐reported previous experiences with the meat alternative, education, income level, and the presence of children are positively associated with consumers’ WTPs, whereas age is negatively associated	Chen, Zhou, et al. (2023)
1169	Exploring psychological barriers to acceptance of cultured meat	Technological neophobia was the main barrier	Krings et al. (2022)
302	To assess whether counter‐messaging strategies, emphasizing the problems of conventional meat production, can enhance consumer acceptance of cultured meat	The counter‐messaging generally improves openness toward cultured meat, regardless of the specific focus. Demographic factors such as age, gender, and dietary habits shape baseline acceptance, whereas psychological variables—particularly perceived consumer effectiveness and limited prior knowledge—are more strongly linked to changes in attitudes	Baum et al. (2022)
376	To investigate Dutch and Finnish consumer attitudes toward plant‐based meat substitutes, cultured meat, and hybrid meat products. To determine how these attitudes relate to consumer attachment to meat, food neophobia, and knowledge of food sustainability	Omnivorous participants tended to be more attached to meat, scored higher on food neophobia, and exhibited less knowledge about food sustainability compared to participants with flexible diets. Meat substitutes and hybrid meat products scored higher, although participants’ willingness to purchase hybrid meat and cultured meat products was significantly lower than their willingness to purchase meat substitutes. Hybrid meat products may be a viable option for reducing meat consumption if properly promoted	van Dijk et al. (2023)
2039	Analyze innovation characteristics in alternative foods: cultivated foods, plant‐based foods, and insect‐based foods	Consumers exhibited significantly higher levels of trust and intention to purchase plant‐based food products, such as plant‐based meat and milk, compared to cultivated food products, including cultured fresh meat, cultured processed meat, cultured seafood, and cellular milk, as well as insect‐based foods	Wang et al. (2024)
942	To observe the attitudinal and behavioral differences between carnivores, people who want to reduce their meat intake, and occasional meat eaters in terms of their meat consumption and attitudes toward future food	People who want to reduce their meat intake find alternatives like cultured meat more appealing	Kemper et al. (2023)

Evidence suggests that acceptance of cultured meat still faces significant barriers, both psychological and cultural. Studies such as those by de Oliveira Padilha et al. (2022) and Sheng et al. (2025) highlight that willingness to consume cultured meat remains moderate, with perceptions of unnaturalness and technophobia (fear or distrust of new technologies) being key factors in consumer hesitation. This phenomenon, known as technological neophobia, was also highlighted by Krings et al. (2022) as a barrier to acceptance.

On the other hand, studies such as those by Chia et al. (2024) and B. Chen, Zhou, et al. (2023) revealed that men and more informed individuals, particularly those concerned with environmental and health issues, tend to show greater acceptance. These results suggest that positive information strategies, emphasizing nutritional, environmental, and safety benefits, can enhance product attractiveness and increase purchase intentions. Regarding willingness to pay, economic and demographic factors are crucial. Studies by B. Chen, Zhou, et al. (2023) and Wang et al. (2024) indicate that individuals with higher income and education are more likely to purchase and consume cultured meat, whereas older individuals demonstrate greater resistance.

According to the studies analyzed in this review, cultured meat has the potential to transform the food sector, but its acceptance depends on overcoming psychological, cultural, and economic barriers through effective strategies. Investments in research, educational campaigns, and reduced production costs are essential to expanding the market and making cultured meat a viable and attractive alternative for consumers. Furthermore, strategic communication initiatives emphasizing concrete benefits, such as sustainability, health, and food safety, are crucial to fostering consumer acceptance.

## 4 Conclusion

This review demonstrates that cultured meat represents an innovative alternative to conventional meat, with significant potential to reduce the environmental impact of livestock farming and advance animal welfare. However, its global viability depends on overcoming technological, nutritional, ethical, and regulatory challenges. Large‐scale implementation requires rigorous evaluations to ensure food safety, with particular attention to mitigating risks from potential chemical and biological contaminants. Nutritionally, the protein and lipid composition of cultured meat requires further improvement to meet consumer demands and achieve a sensory profile comparable to traditional meat. Furthermore, consumer acceptance remains a major obstacle to market consolidation, strongly influenced by cultural, economic, and psychological factors. The groups most likely to adopt cultured meat include men, younger individuals, and those with higher education and income levels, as well as health‐conscious consumers and those following meat‐reduced diets. Ultimately, the consolidation of cultured meat as a viable commercial alternative depends on joint efforts to establish an international regulatory consensus that balances safety, sustainability, and the complex ethical and cultural considerations inherent in the production of innovative foods. Future advances in bioprocess engineering, cost reduction, and transparent communication will determine whether cultured meat transitions from a technological novelty to a sustainable global food solution.

## Author Contributions

**Ana Carolina Agne Ferreira Zão**: conceptualization, writing – original draft, data curation. **Wesclen Vilar Nogueira**: writing – review and editing. **Filipe Soares Rondan**: writing – review and editing. **Priscila Tessmer Scaglioni**: conceptualization, writing – review and editing, supervision, methodology.

## Funding

The authors have nothing to report.

## Conflicts of Interest

The authors declare no conflicts of interest.
